# The Study of EDLC Device with High Electrochemical Performance Fabricated from Proton Ion Conducting PVA-Based Polymer Composite Electrolytes Plasticized with Glycerol

**DOI:** 10.3390/polym12091896

**Published:** 2020-08-23

**Authors:** Mohamad A. Brza, Shujahadeen B. Aziz, Hazleen Anuar, Elham M. A. Dannoun, Fathilah Ali, Rebar T. Abdulwahid, Shakhawan Al-Zangana, Mohd F.Z. Kadir

**Affiliations:** 1 Department of Manufacturing and Materials Engineering, Faculty of Engineering, International Islamic University of Malaysia, Kuala Lumpur, Gombak 53100, Malaysia; mohamad.brza@gmail.com (M.A.B.); hazleen@iium.edu.my (H.A.); 2 Advanced Polymeric Materials Research Lab., Department of Physics, College of Science, University of Sulaimani, Qlyasan Street, Sulaimani 46001, Iraq; rebar.abdulwahid@univsul.edu.iq; 3 Department of Civil Engineering, College of Engineering, Komar University of Science and Technology, Kurdistan Regional Government, Sulaimani 46001, Iraq; 4 Associate Director of General Science Department, Woman Campus, Prince Sultan University, P. O. Box 66833, Riyadh 11586, Saudi Arabia. elhamdannoun1977@gmail.com; 5 Department of Biotechnology Engineering, Faculty of Engineering, International Islamic University of Malaysia, Kuala Lumpur, Gombak 53100, Malaysia; fathilah@iium.edu.my; 6 Department of Physics, College of Education, University of Sulaimani, Old Campus, Kurdistan Regional Government, Sulaimani 46001, Iraq; 7 Department of Physics, College of Education, University of Garmian, Kurdistan Regional Government-Iraq, Kalar 46021, Iraq; shakhawan.al-zangana@garmian.edu.krd; 8 Centre for Foundation Studies in Science, University of Malaya, Kuala Lumpur 50603, Malaysia; mfzkadir@um.edu.my

**Keywords:** polymer composite electrolyte, ammonium slat, metal complex, glycerol plasticizer, electrical properties, electrochemical double-layer capacitor

## Abstract

In the present work, a novel polymer composite electrolytes (PCEs) based on poly(vinyl alcohol) (PVA): ammonium thiocyanate (NH_4_SCN): Cd(II)-complex plasticized with glycerol (Gly) are prepared by solution cast technique. The film structure was examined by XRD and FTIR routes. The utmost ambient temperature DC ionic conductivity (*σ_DC_*) of 2.01 × 10^−3^ S cm^−1^ is achieved. The film morphology was studied by field emission scanning electron microscopy (FESEM). The trend of *σ_DC_* is further confirmed with investigation of dielectric properties. Transference numbers of ions (*t_ion_*) and electrons (*t_el_*) are specified to be 0.96 and 0.04, respectively. Linear sweep voltammetry (LSV) displayed that the PCE potential window is 2.1 V. The desired mixture of activated carbon (AC) and carbon black was used to fabricate the electrodes of the EDLC. Cyclic voltammetry (CV) was carried out by sandwiching the PCEs between two carbon-based electrodes, and it revealed an almost rectangular shape. The EDLC exhibited specific capacitance, energy density, and equivalent series resistance with average of 160.07F/g, 18.01Wh/kg, and 51.05Ω, respectively, within 450 cycles. The EDLC demonstrated the initial power density as 4.065 × 10^3^ W/Kg.

## 1. Introduction

Lately, proton conducting polymer-based electrolyte is emerged like an important subject for uses in energy storage devices. Polymer electrolytes (PEs) with proton (H^+^) as a carrier species were used in the application of electrochemical devices [1,2]. Proton-conducting PEs with powerful inorganic acid for instance sulfuric acid (H_2_SO_4_) [3] as well as phosphoric acid (H_3_PO_4_) [4] have drawbacks because of the chemical degradation with a weak mechanical integrity. For this reason, ammonium salts have been employed as excellent candidates instead of inorganic acids as a source for H^+^ ions in PEs [5,6]. The small lattice energy ammonium salts could be employed as fillers to get a novel PEs with large DC ionic conductivity (*σ_DC_*). Ammonium thiocyanate (NH_4_SCN) possesses 605 kJ/mol lattice energy, thus simply dissociates into anions and cations, and has an anionic (SCN^−^) size of 250 pm [7,8]. The PEs fabrication with large *σ_DC_* at ambient temperature is one of the key aims in the research of PEs. The improvement in *σ_DC_* values in complexes of polymers and salts open the gate toward the electrochemical devices fabrication such as photochemical cells [9], supercapacitors (Scs) [10], proton batteries [11] as well as fuel cells [12]. Amongst the presented polymers, poly(vinyl alcohol), PVA, is one of the main biodegradable and biocompatible polymers with non-toxic property. Hema et al. documented the maximum *σ_DC_* of 10^−5^ S cm^−1^ for the PE of PVA–NH_4_Cl [13].

The inclusion of cadmium(II)-complex (Cd(II)-complex) to the PE was attempted to improve the *σ_DC_* and amorphous phase. In our previous report, it was observed that insertion of Cu(II)–complex to PVA expanded the amorphous structure [14].It is well reported that amorphous phase is crucial for ion transport mechanism. The interaction between Cu(II)-complex and the PVA host matrix enrich the amorphous region, which in turn rise the conductivity of the polymer [14]. In the current work, glycerol (Gly) is also attempted as plasticizer to further increase *σ_DC_*. Pawlicka et al. [15] documented an increase in *σ_DC_* from around 10^−8^ to10^−4^ S cm^−1^ by addition of Gly to their system of PE. Gly with high dielectric constant weakens the attraction force amongst the anions and cations of the salts, thus extra mobile ions would be offered [16]. The plasticizer addition can enhance the PE amorphous structure [17].

In 1989, the first electrical double-layer capacitor (EDLC), also named the Sc has been fabricated [18,19,20,21]. The EDLC could be employed in several uses, e.g., in hybrid or electric vehicles like the energy storage device. EDLC is an electrochemical device in which the mechanism of the stored energy happens as a result of the buildup of ions at the interfaces between the PEs and blocking electrodes [22]. Activated carbon (AC) is used in this study as an electrode material because of its high conductivity, chemical stability, large surface area (>1000 m^2^ g^−1^), cost feasibility, and high porosity (>2 nm pore width) [23,24]. Bigger surface area offers extra electrosorption of NH_4_^+^ and SCN^-^ ions, therefore offering larger capacitance. AC electrodes porosity is also important in the fabrication of EDLC with larger capacitance. Heimbockel et al. [25] documented that the capacitance improved when the pore size enlarged. AC has reasonable big pore size in the range between 20 and 200 Å. Big pore size permits extra solvated ions to come in and go through the process of adsorption. EDLCs possess the same electrodes, extended lifetimes, large cyclabilities, as well as great power density [26].

There are some documents that used polymer composite electrolytes (PCEs) in the synthesis of EDLC [27,28]. However, no study has been reported in literature regarding the effect of Cd(II)-complex on the EDLC performance. Liew [27] had fabricated EDLC based on poly(acrylic acid) (PAA):lithium bis(trifluoromethanesulfonyl)imide (LiTFSI):barium titanate (BaTiO_3_). They showed that the incorporation of BaTiO_3_ into PAA:LiTFSI system provides the *σ_DC_* of 5 × 10^−4^ S cm^−1^ and specific capacitance (*C_d_*) of 34.22 F g^−1^. However, the *C_d_* of the PCE is still low and the inclusion of BaTiO_3_didnot significantly enhanced the electrochemical performances of the EDLC. The goal of this work is to improve the amorphousness of PVA through the addition of Cd(II)-complex. The Gly plasticizer further extended the structure of amorphous and dissociated extra ions to contribute in *σ_DC_*. The high *σ_DC_* gained in this study is a guarantee for application in electrochemical devices, e.g., EDLC device with high performance.

## 2. Materials and Methods

### 2.1. Materials

Sigma-Aldrich (Kuala Lumpur, Malaysia) offered poly(vinyl alcohol) (PVA) powder material (average molecular weight = 85,000–124,000), cadmium(II) nitrate (molecular weight = 236.42 g/mol), and Gly (molecular weight = 92.09382g/mol). Ammonium thiocyanate (NH_4_SCN) (molecular weight = 76.12g/mol), N-Methyl-2-pyrrolidone (NMP) (molecular weight = 99.13 g/mol), and carbon black (molecular weight = 12.01 g/mol) were procured, respectively, from HmbG chemicals, EMPLURA, and Timcal. Polyvinylidene fluoride (PVdF) (average molecular weight =~534,000 by GPC) and activated carbon (RP20) (molecular weight = 12.01 g/mol) were procured from magna value.

### 2.2. Electrolyte Preparation

Here, 50 wt.% PVA was dissolved in distilled water (40 mL). The solution was mixed via magnetic stirrer at 80 °C for roughly 60 min for the PVA solution creation. The PVA solutions were then cooled down to ambient temperature. The PVA polymer solutions were inserted with 50 wt.% NH_4_SCN. The solutions were mixed incessantly via magnetic stirrer at room temperature till NH_4_SCN salt was completely dissolved in the PVA solutions. Consequently, PVA:NH_4_SCN were inserted with 10 mL Cd(II)-complex. The Cd(II)-complex was obtained through adding a dissolved Cd(NO_3_)_2_ into the extract tea leaf solution at 80 °C. This mixture was stirred for nearly 10 min until the color of solution changed from dark-colored to yellow and precipitation created as a cloud at the beaker bottom, which confirms the Cd(II)-complex formation. The detailed procedure of synthesis of Cd(II)-complex is the same as that described in the previous report in Materials and Methods section in ref. [14]. Afterward, the solutions were merged and stirred to guarantee the proper dispersion of Cd(II)-complex in the solution. Lastly, the PVA:NH_4_SCN:Cd(II)-complex were plasticized with 10, 20, 30, and 40 wt.% Gly and then the prepared samples were coded as PNCG-1, PNCG-2, PNCG-3, and PNCG-4, respectively. Subsequently, the plasticized solutions were stirred until a homogeneous solution was accomplished and then conserved in the plastic Petri dishes and left for drying at room temperature. For better drying, the fabricated films were conserved in a desiccator that includedsilica gel prior to examination.

### 2.3. Characterization Techniques

#### 2.3.1. X-ray Diffraction (XRD) Measurements

Pattern of X-ray diffraction (XRD) have been provided by Empyrean X-ray Diffractometer, (PANalytical, Netherland) with 40 KV applied voltage and 40 mA applied current. The PCE films and Cd(II)-complex were enlightened with a monochromatic CuKα X-radiation at 1.5406 Å wavelength with the 2θ glancing angles settled between 10° and 80° with 0.1° in step size.

#### 2.3.2. Fourier-Transform Infrared Spectroscopy (FTIR)

To study the PVA film and PVA:NH_4_SCN:Cd(II)-complex:Gly films, Fourier-transform infrared (FTIR) spectrophotometer (Thermo Scientific, Nicolet iS10) was settled in the wavenumber between 4000 and 450 cm^−1^ with resolution of 2 cm^−1^.

#### 2.3.3. Field Emission Scanning Electron Microscopy (FESEM)

A Hitachi SU8220 has been applied to conduct field emission scanning electron microscopy (FESEM) at 500× magnification. FESEM images were captured to examine the films morphology.

#### 2.3.4. Electrochemical Impedance Spectroscopy (EIS)

The impedance spectra of pure PVA and PVA:NH_4_SCN:Cd(II)-complex:Gly have been provided using electrochemical impedance spectroscopy (EIS) [3532-50 LCR HiTESTER (HIOKI)] in the frequencies between 50 and 5,000,000 Hz. The synthesized films have been cut to small circles (diameter = 2cm) and then sited between stainless steel (SS) electrodes due to pressure of a spring. The cell has been merged to a computer program to provide real (Z^’^) and imaginary (Z^”^) parts of the spectra of the complex impedance (Z^*^).

### 2.4. Electrolyte Characterization

#### Ionic Transference Number Analysis and Linear Sweep Voltammetry

Transference number (TNM) of ionic (*t_ion_*) and electronic (*t_el_*) were determined. The cell preparation was SS| highest conducting PCE (PNCG-4)| SS. The cell was coupled to the V&A Instrument Digital DC Power Supply DP3003 as well as UNI-T UT803 multimeter. The voltage applied was 0.2 V where the cell was polarized with time at room temperature.

Before EDLC device fabrication, linear sweep voltammetry (LSV) investigation has been met to observe the potential stability of the PCE. A 10 mV s^−1^ scan rate was employed between 0 and 2.5 V at room temperature. The cell was linked to the working electrode, reference electrode, and counter electrode due to potentiostat of Digi-IVY DY2300. When potential between working electrode and counter electrode was found linearly sweep, the alteration in the current values at the working electrode was recorded.

### 2.5. Electrode Preparation

Planetary ball miller (XQM-0.4) was used to merge 3.25 g of activated carbon (AC) and 0.25 g of carbon black, for roughly 20 min at 500 rpm. Six balls of metal were incorporated to a chamber with the above powders together. Besides, 0.5 g of polyvinylidene fluoride (PVdF) was doped in the 15 mL of solvent N-methyl pyrrolidone (NMP) and then stirred for nearly 60 min. The powders were incorporated to the solution of PVdF-NMP and then stirred for 120 min until there was the emergence of thick black homogenous solution. An aluminum foil was cleansed using acetone, and after that, the synthesized homogenous solution was covered on it using the doctor blade technique. Afterwards, the manufactured electrodes were dried-out inside an oven at 60 °C, and accordingly, the dried-out electrodes were inserted in a desiccator with silica gel for advance drying.

### 2.6. EDLC Characterization

The electrodes were cut into small circles (area = 2.01 cm^2^). The highest conducting PCE (PNCG-4) was introduced between the two AC electrodes and then introduced in a coin cell of CR2032. Followed by, this CR2032 was sited in a Teflon case.

Potentiostat of Digi-IVY DY2300 was performed to conduct EDLC cyclic voltammetry (CV). The used potential was from 0 to 0.9 V with a number of scan rates. The EDLC was introduced with different scan rates of 5, 10, 20, 50, and 100 mV s^−1^.

The rechargeability of EDLC was examined by NEWARE battery cycler for 450 cycles at 0.5 mA cm^−2^ current density. The surrounding for CV and charge-discharge of the EDLC analysis was settled at nearly 25 °C and ~50% relative humidity.

## 3. Results and Discussion

### 3.1. XRD Analysis

**Figure 1:**
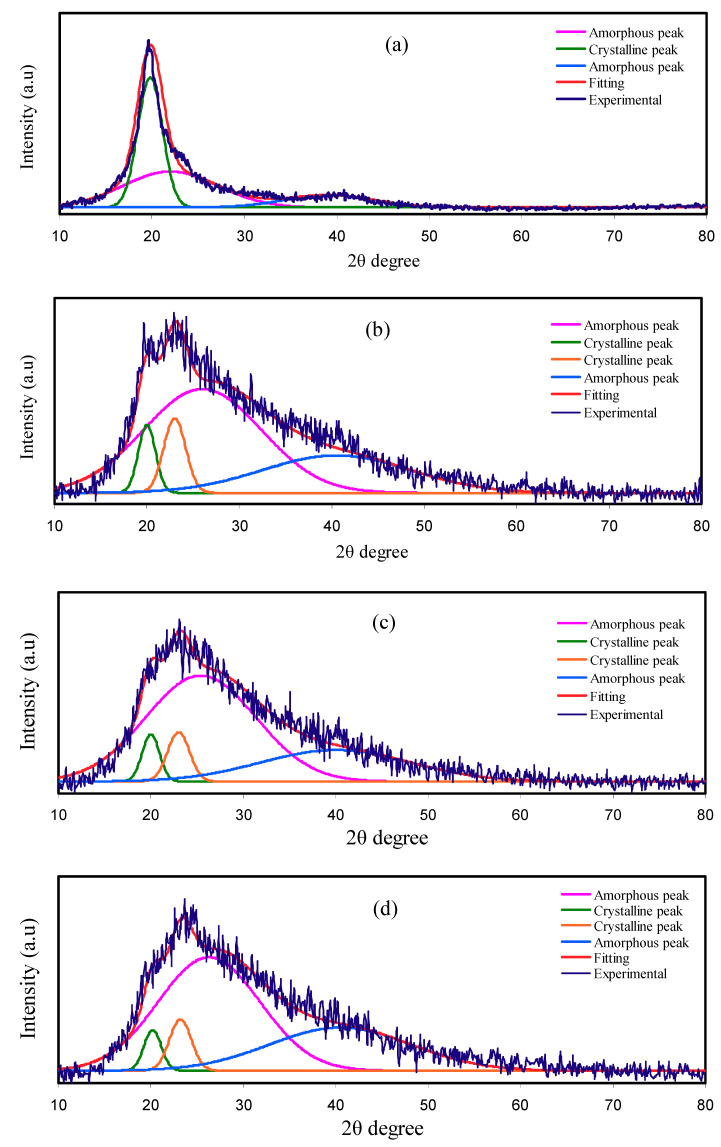

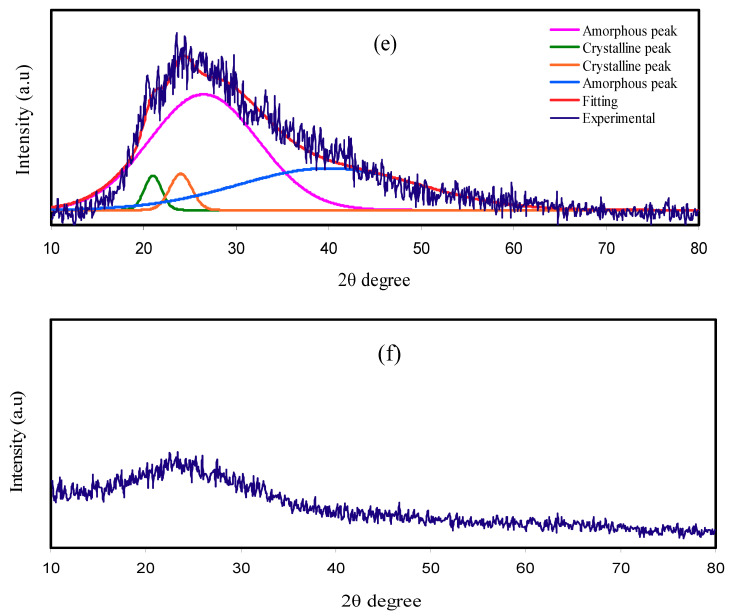
Deconvoluted XRD spectra for (**a**) pure PVA, (**b**) PNCG-1, (**c**) PNCG -2, (**d**) PNCG-3, and (**e**) PNCG-4 films, and (**f**) synthesized Cd(II)-complex.

The trend of *σ_DC_* is confirmed due to the investigation of XRD, where the dissimilarity in amorphous structure of the PEs is detected. The deconvoluted XRD spectra correlated with pure PVA, PVA:NH_4_SCN:Cd(II)-complex:Gly, and synthesized Cd(II)-complex are observed in Figure 1. The peaks at roughly 2θ = 20°and 40° that have been discerned in the pure PVA XRD spectrum are correlated in earlier documents with the PVA crystalline domains [5,7]. It has been observed that these two peaks stayed in the spectra of incorporated PVA, although their intensities were decreased. More specifically, the 2θ = 40°peak intensity is significantly decreased, though the peak at 2θ = 20°is expanded. The amorphous structure enhancement is noted in the wideness increase and intensity reduction of the peak at 2θ = 20° [29,30].

The salt addition to the polymer is likely to enhance the amorphous structure of PE. The connection between *σ_DC_* and degree of amorphous nature is correlated to the superior ionic mobility and diffusivity of ions within the amorphous structure owing to small barriers of energy. In amorphous structure, backbones of polymer are extra flexible as well as possess larger segmental movement of chains. The segments movement in the amorphous structure enhances ionic movement through creating and breaking the solvated ions coordination sphere with offering more free space or volume in which the ions can diffuse due to the electrical field (EF) effect [31,32]. Nonetheless, there is a reduction in the *σ_DC_* at uppermost concentration of salt in the previous study because of the reduction of free ions with the recrystallization increment [33]. To overcome these issues, the addition of Cd(II)–complex and Gly to the PE were attempted to improve the structure of amorphous PE and *σ_DC_*. The nature of amorphous PE in the electrolyte system was expanded with the insertion of metal complexes and Gly [14,17]. The Gly incorporation into the system of polymer and salt assists salt dissociation, which consequently reduces the salt recrystallization occurrence. Additionally, the plasticizers can offer alternating pathways for conduction of ions, thus aiding the polymers in order to accommodate salts. These mechanisms raise the amorphous structure in the PEs, afterward enhancing the electrolyte *σ_DC_* [33].

The XRD pattern for Cd(II) complex (see Figure 1f) shows an overall amorphous nature since there is no crystalline peaks throughout the 2θ degrees. The Cd (II) metal complex interacts with the functional groups in the polymer and disrupt hydrogen bonding among polymer chains. This nearly full amorphous nature of Cd(II) complex will improve the overall amorphous phase of the prepared samples, which acts as a pathway for ion conduction and enhance the conductivity. Similarly, in the previous study, it was observed that when Cu(II)-complex was added to the PVA, the amorphous structure has been improved, which consequently causes a decrease in the intensity of XRD spectra [14]. Previous study indicated that the XRD pattern of the Cu(II)-complex is almost amorphous, therefore, crystalline peaks were not detected within the entire 2θ degrees. Noticeably, a hump only was observed from 2θ = 20° to 30° [14]. XRD analysis results confirm that complexation takes place between the PVA and electrolyte components. In addition, the nonappearance of any peaks linked to pure PVA indicates the whole NH_4_SCN salt dissociation in the PE [34].

The technique of deconvolution for the XRD spectra has been employed so as to get the probable amorphous peaks as well as crystalline peaks [35]. The degree of crystallinity (Xc) has been gained using the deconvoluted XRD spectra as seen in Figure 1. The wide and large peaks denote the peaks of amorphous, whereas the narrow, sharp, and small peaks indicate the peaks of crystalline. The degree of crystallinity for pure PVA is 41.68 and significantly reduced upon the inclusion of Cd(II)-complex and Gly. Hence, the amorphous structure of the system of polymer and salt is expanded with the insertion of Cd(II)–complex and Gly. As demonstrated in Figure 1, the peaks of crystalline in PNCG-1are smaller as well as less sharp with the Gly increment (see Figure 1c–e). Inclusion of 40 wt% Gly produces smaller peaks of crystalline as indicated in the XRD spectrum of PNCG-4 (see Figure 1e). The degree of crystallinity for pure PVA and PCEs have been gained with Equation (1) and sorted in Table 1. The degree of crystallinity of PNCG-1 PCE is 13.64. The smallest degree of crystallinity, i.e., 6.47, was gained for PNCG-4. This means that PNCG-4 is mainly amorphous PCE in the current work. The *σ_DC_* values obey the degree of crystallinity trend [36].

(1) $$X_{C}=\;\frac{A_{C}}{A_{T}}\;\times \;100\%$$

where *A_T_* and *A_C_* denote the areas of total amorphous and crystalline peaks, respectively, that have been obtained by means of the method of deconvolution using OriginPro software. The mode of Gaussian function has been employed to fit the XRD spectra.

### 3.2. FTIR Analysis

**Figure 2:**
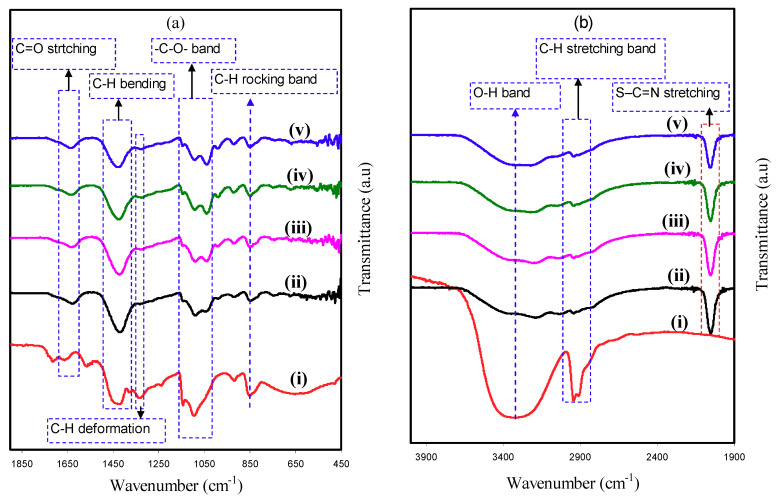
FTIR spectra for (**i**) pure PVA, (**ii**) PNCG-1, (**iii**) PNCG -2, (**iv**) PNCG-3, and (**v**) PNCG-4 in the region (**a**) 450–1900 cm^−1^ and (**b**) 1900–4000 cm^−1^.

The spectra of FTIR for pure PVA and PVA:NH_4_SCN:Cd(II)-complex:Gly are indicated in Figure 2a,b. The following modifications in the spectral characteristics have been detected, after that the spectra of doped PVA with the pure PVA are compared. In the meantime, O–H stretching vibration of hydroxyl groups is related with the extensive and robust absorption peak at 3317 cm^−1^ [37]. This band shows a high intensity, almost certainly owing to the robust intra- and intermolecular hydrogen bond [5]. Moreover, this band moves and exhibits a drastic decline in intensity for the incorporated PVA films as a result of its interaction with NH_4_SCN salt, Cd(II)-complex, and Gly. C–H asymmetric stretching vibration was interrelated with a band at 2913 cm^−1^ [37] that shifts and declines noticeably in the case of incorporated PVA films.

It is recognized that in NH_4_SCN salt-incorporated PE, the carrier species is H^+^ [38]. The new strong and intense peak observed at 2045 cm^−1^ is certified to aromatic S–C=N stretching of SCN^−^ anion group of NH_4_SCN. This band shifts and displays a notable reduction in intensity at the highest Gly amount as a result of its complexation with the functional groups in the PVA (see Figure 2b) [7,34]. In the tetrahedral ammonium ion NH_4_^+^, as one of four H^+^ linked to nitrogen atom is not strongly bound, H^+^ can transfer to each coordinating site in the PVA. The change in the place of the peak and the emergence of the new peak in the incorporated PVA system demonstrates the creation of complex between the PVA and the NH_4_SCN [7]. In addition, the interaction between functional groups in PVA and Cu(II)–complex was revealed in the previous study [14]. The plasticizer insertion also aids ion dissociation to take place; for that reason, more ions are offered to interact with the functional groups in the PVA [39].

C=O stretching of acetate group was the cause for the peak at 1643 cm^−1^ in pure PVA [40], while in the incorporated PVA films, it is deviated to smaller wavenumbers. The C–H bending vibration of CH_2_ wagging is the cause of absorption peak at 1415 cm^−1^ in pure PVA, while C–H deformation vibration is the cause of absorption peak at 1319 cm^−1^ in pure PVA (see Figure 2a) [40]. It is, therefore, obvious that the incorporated PVA samples were interrelated with shifting of these couple of peaks with a great drop in the intensity of these peaks. These variations in the spectra of FTIR clearly suggest the interaction of PVA functional groups with the electrolyte components (see Figure 2a,b).

Furthermore, the –C–O– stretching vibration in pure PVA is recognized through the peak at 1082 cm^−1^ [41], which is shifted and reduces some of its intensity in the doped films with Cd(II)-complex and Gly, as shown in Figure 2a. C–H rocking of pure PVA is considered the reason of the absorption peak at 838 cm^−1^ (see Figure 2a) [5]. In the case of incorporated PVA films, this peak shifted and its intensity decreased, whereas addition of 40 wt.% Gly caused a significant decrease in its intensity. These results are tabulated in Table 2.

### 3.3. Morphological Study

**Figure 3:**
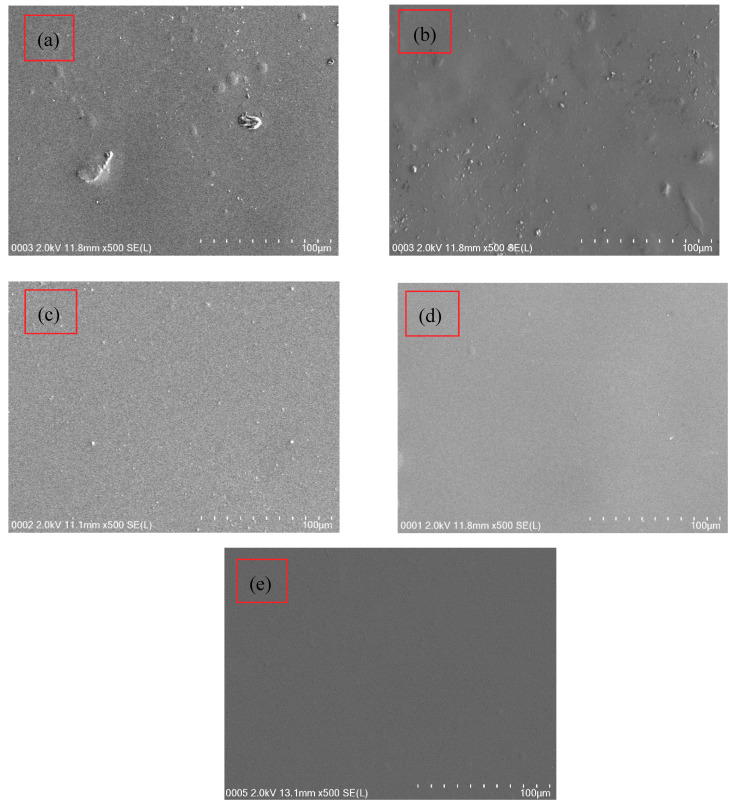
FESEM images for (**a**) PNCG-1, (**b**) PNCG-2, (**c**) PNCG-3, (**d**) PNCG-4, and (**e**) pure PVA film.

To support the outcomes of XRD, field emission scanning electron microscopy (FESEM) images were received at 500× magnification for the fabricated PCEs. Figure 3a–e shows the FESEM for PVA:NH_4_SCN:Cd(II)-complex:Gly and pure PVA. When Cd(II)-complex with 10 and 20 wt.% Gly have been inserted to the PE system, some salts emerged to protrude throughout the films surface as demonstrated in Figure 3a,b compared to the pure PVA (see Figure 3e). The developed protruded salts in Figure 3a,b are ascribed to the creation of ion aggregation, which consequently decreases the σ_DC_ [11]. From the outcomes of FESEM, it is straightforward to comprehend that the intense crystalline peaks achieved from the deconvoluted XRD spectrum of the PNCG-1 system (see Figure 1a) are associated to the protruded salt.

Morphologically, the PNCG-3 and PNCG-4 images (see Figure 3c,d) possessed a uniform surface and it has been detected to be homogenous and smooth lacking any separation in phase. These outcomes indicate that PVA polymer can dissolve NH_4_SCN salt with rising amount of Gly. It is worth mentioning that electrolytes with smooth surface are interconnected, which allow the ions to travel freely and as a consequence resulting in a higher *σ_DC_* value [42]. Arof and coworkers [43] fabricated CS:PVA:xNH_4_NO_3_ SPEs and represented a fall in *σ_DC_* at a higher amount of salt. They detected agglomeration of ions to protrude from the surface. In the current study, the outcomes display that there is a novelty in the PCE preparation as a new and simple technique to get large *σ_DC_* and high electrochemical performance EDLC. From the FTIR results, the interactions of the functional groups in PVA and electrolyte components were described. It is explicit that the films surface morphology has been discovered to be approximately smooth lacking any observed perceptible protruded particles at large amounts of doped Gly.

It was documented that challenges still persisted for getting clearly defined morphologies as well as enhancing characteristics of ion transport in PEs. It is thought that large *σ_DC_* is connected with the homogenous and smooth emergences of the films, meaning that it is interconnected to the amorphous structure of the samples [44]. The addition of Cd(II)-complex and Gly enhanced the amorphous and *σ_DC_*. The addition of Cu(II)-complex into PVA enhanced the amorphous structure as indicated in the previous study [14]. Similarly, the PCEs uniform surface in Figure 3c,d reveals the good dispersion of Cd(II)-complex. The small white spots emerging on the PCEs surface are ascribed to the Cd(II)-complex. In fact, the complexation between the PVA and electrolytes components is satisfied as a result of the smooth uniform surface morphology of the PCEs (see Figure 3c,d).

### 3.4. Impedance Analysis

**Figure 4:**
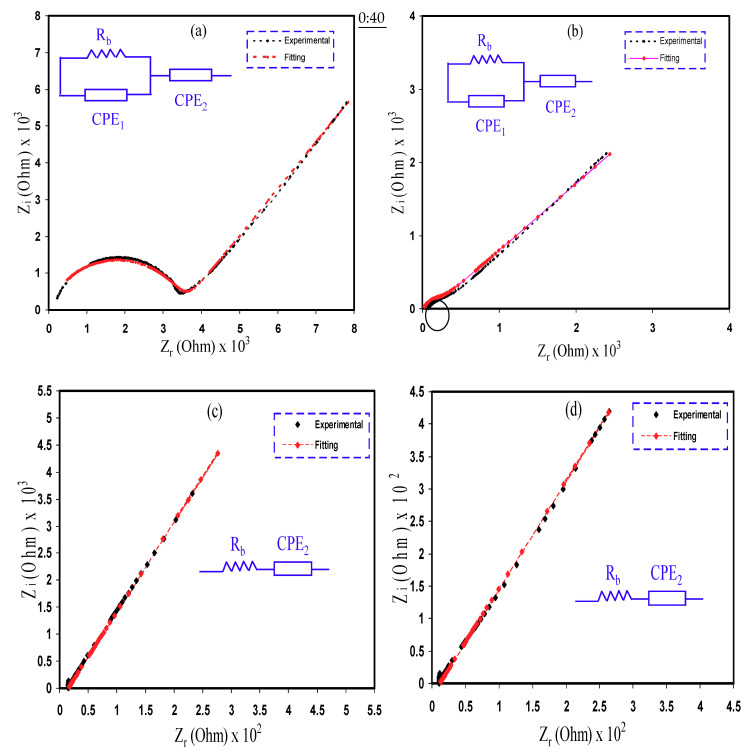
Electrochemical impedance spectroscopy (EIS) plots for (**a**) PNCG-1, (**b**) PNCG-2, (**c**) PNCG-3, and (**d**) PNCG-4 electrolytes.

The Cole–Cole plots for the PCE films at ambient temperature are displayed in Figure 4. The values of bulk resistance (R_b_) are found due to the interaction between the semicircle curve and the real axis at the region of low frequencies. On inclusion of 10 wt.% and 20 wt.% Gly (i.e., PNCG-1 and PNCG-2 electrolytes, respectively), the Cole–Cole plots include a semicircle at the region of high frequencies as well as a tail or spike at the region of low frequencies as displayed in Figure 4a,b. The semicircle is correlated to the conduction of ions at the bulk of the PEs [45]. As documented by Malathi et al. [31], the σ_DC_ at the bulk is ascribed to the parallel connection of *R_b_* and capacitance at the bulk of the PEs. The tail or spike is related to the electrode polarization impact that is a feature of diffusion mechanism [45]. The semicircle is diminished as the amount of Gly increased to 20 wt.% (i.e., PNCG-2). The values of *R_b_* decreased as the amount of Gly raised up to 40 wt.% (i.e., PNCG-4). The spike was only demonstrated by the other samples (see Figure 4c,d).

The method of electrical equivalent circuit (EEC) has been performed for the inspection of electrochemical impedance spectroscopy (EIS), since the method is effortless, rapid, and manufactures a total picture of the PE [46]. The Nyquist plot for the PCEs are deduced with regard to the equivalent circuit (EC) consisting of *R_b_* for the carrier species in the PCEs with a couple of constant phase elements (CPE), which are CPE_1_ and CPE_2_, as demonstrated in the inserts of Figure 4. The high frequencies region reveals the connection of *R_b_* and CPE_1_ in parallel, whereas the low frequencies region indicates CPE_2_, distinctly, the fabricated electrochemical double-layer capacitance between electrodes and PEs. The CPE name is regularly employed in the EC rather than ideal capacitor in real system.

The impedance of *Z_CPE_* is written as [36,47]:

(2) $$Z_{CPE}=\;\frac{1}{\;C\omega^{p}}\left[\cos\left[\frac{\pi p}{2}\right]-i\sin\left[\frac{\pi p}{2}\right]\right]$$

where *C* stands for the CPE capacitance, *ω* stands for the angular frequency, and *P* is interrelated to the departure of the EIS plots from the vertical axis. Here, the parts of real (*Z_r_*) and imaginary (*Z_i_*) of complex impedance (Z^*^) interrelated with the EC (insert of Figure 4a,b) are demonstrated as:

(3) $$Z_{r}=\;\frac{R_{b}{}^{2}C_{1}\omega^{p1}\cos\left[\frac{\pi p_{1}}{2}\right]+R_{b}}{2R_{b}C_{1}\omega^{p1}\cos\left[\frac{\pi p_{1}}{2}\right]+R_{b}{}^{2}C_{1}{}^{2}\omega^{2p1}+1}+\frac{\cos\left[\frac{\pi p_{2}}{2}\right]}{C_{2}\omega^{p2}}$$

(4) $$Z_{i}=\;\frac{R_{b}{}^{2}C_{1}\omega^{p1}\sin\left[\frac{\pi p_{1}}{2}\right]+R_{b}}{2R_{b}C_{1}\omega^{p1}\cos\left[\frac{\pi p_{1}}{2}\right]+R_{b}{}^{2}C_{1}{}^{2}\omega^{2p1}+1}+\frac{\sin\left[\frac{\pi p_{2}}{2}\right]}{C_{2}\omega^{p2}}$$

where *C*_1_ stands for the CPE_1_ capacitance at the bulk of the Pes and *C*_2_ stands for the CPE_2_ capacitance at the electrode–electrolyte interface.

Here, the values of *Z_r_* and *Z_i_* of Z^*^ correlated with the EC (insert of Figure 4c,d) are expressed as:

(5) $$Z_{r}=\;R+\frac{\cos\left[\frac{\pi p_{2}}{2}\right]}{C_{2}\omega^{p2}}$$

(6) $$Z_{i}=\;\frac{\sin\left[\frac{\pi p_{2}}{2}\right]}{C_{2}\omega^{p2}}$$

Table 3 signifies the fitting parameters in the EEC.

It is explicit that the capacitance (*C*) values are larger at the area of low frequencies than at the area of high frequencies that verifies the equation below:

(7) $$C=\frac{\varepsilon_{o}\varepsilon_{r}A}{d}$$

where *ε_o_* stands for permittivity in vacuum and *ε_r_* stands for dielectric constant. Earlier documents [5,36] have exhibited that *ε_r_* values of PEs declines with growing frequency and consequently declines the capacitance. The capacitance values are also seen to rise with increasing the amount of Gly. The *ε_r_* outcomes in this study will be discussed at the next section. Plasticizer insertion to the PE increases the free ions number, and thus increases the *ε_r_* value [48]. From Equation (7), growing *ε_r_* will raise the capacitance values.

The addition of Cd(II)–complex and Gly to the PE improves its amorphous structure, which in turn increases the *σ_DC_* and enhances the EDLC performance. Rangasamy et al. [32] thought that the increase in the amorphous structure of the PE increases the ions mobility by creating more free volume in the PE system. This results in growing of the segmental motion of the polymer chains, because of the rise in the polymer chains flexibility. Consequently, the *σ_DC_* in the PE can be improved. Lim et al. [28] had synthesized the PCE based on PVA-LiClO_4_ with TiO_2_ insertion. The author obtained *σ_DC_* of 1.3 × 10^−4^ S cm^–1^ and applied the PE on the EDLC cells. This indicates that PCE is possible to be employed like an electrolyte supplier in the EDLC fabrication. By taking the value *s* of *R_b_* and the PCEs dimensions, the *σ_DC_* of the PCEs are calculated using Equation (8)

(8) $$\sigma_{dc}=\;\left[\frac{1}{R_{b}}\right]\times \left[\frac{t}{A}\right]$$

where *t* stands for the PCEs thickness and *A* stands for the SS electrodes area. The *σ_DC_* of the PCEs is sorted in Table 4. According to the previous report, the *σ_DC_* of 10^−4^ S cm^−1^ is satisfactory to utilize in electrochemical devices [49].

### 3.5. Dielectric Studies

The *σ_DC_* is further described by investigation of dielectric properties. The real (*ε’*) and imaginary (*ε”*) parts of complex dielectric constant (ε*) have been achieved using Equations (9) and (10):

(9) $$\varepsilon^{\prime }=\;\frac{Z^{\prime \prime }}{\omega C_{o}\;\left(Z^{\prime 2}+\;Z^{\prime \prime 2}\right)}$$

(10) $$\varepsilon^{\prime \prime }=\frac{Z^{\prime }}{\omega C_{o}\;\left(Z^{\prime 2}+\;Z^{\prime \prime 2}\right)}$$

where *ω* stands for the radial frequency, *C_o_* stands for the capacitance in free space, *Z’* denotes the impedance real part, and *Z’’* stands for the impedance imaginary part. Dielectric constant (*ε’*) is defined as the charge storage, whereas the loss in energy to transport ions when the polarity of applied EF is reversed quickly is named as dielectric loss (*ε’’*) [50].

**Figure 5:**
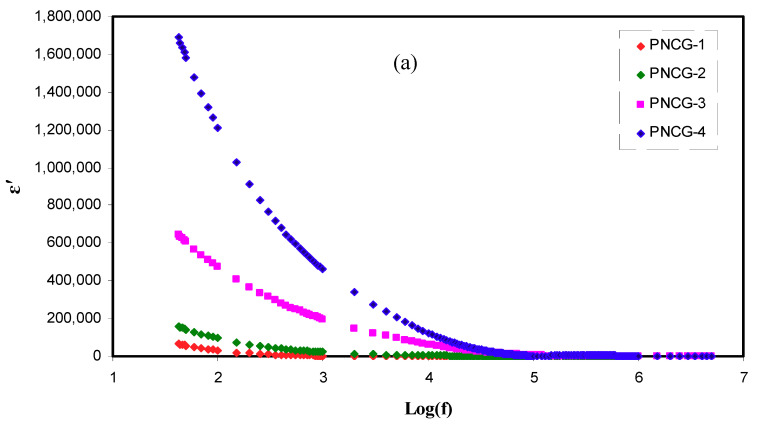

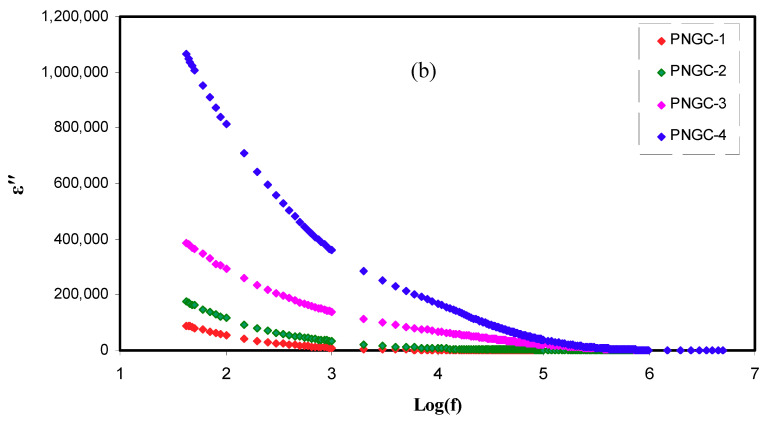
Complex dielectric constant plot (**a**) ε’ versus log (f) and (**b**) ε’’ versus log (f) for all polymer composite electrolytes(PCEs).

Figure 5a,b shows the dependence of frequency of *ε’* and *ε’’* for PCEs at room temperature. Relaxation peaks are not seen, demonstrating that the values of *ε’* would be employed like an indicative to reveal that the growing *σ_DC_* is principally due to the increasing free ions [51]. In the figures, the rising amount of Gly improves the *ε’* and *ε’’* values. When the amount of Gly is increased, the stored charge in the PCEs is more enhanced, signifying the improved ions number density. In Figure 5a,b, PNCG-4 electrolyte possesses the uppermost values of *ε’* as well as *ε’’*. These results were satisfied by the values of *σ_DC_* sorted in Table 4. As reported by Shukur et al. [52], the plasticized PE with the largest *σ_DC_* shows the largest value of *ε’*. The Cd(II)-complex insertion enhanced the structure of amorphous, and the Gly inclusion increases the salt dissociation degree. Consequently, the mobility of ions rises by declining the potential barrier for the ions movement, resulting in the reduction of coordination between anions and cations in the salt [53].

As documented by Shukur et al. [52], both *ε’* and *ε’’* values at the high region of frequencies increased as the Gly amount raised up to 30 wt.% and declined with the insertion of 35 and 40 wt.% Gly. The plasticizer insertion improves the dissociation of ions, thus increasing the number of mobile ions [39]. In this circumstance, the *σ_DC_* grows. The *ε’* and *ε’’* decrement at 35 and 40 wt% Gly in ref. [52] is caused by the recombination of ions. Previous documents displayed that the pattern of conductivity is agreed with the outcomes of dielectric constant [36,52]. In the present work, in all amounts of Gly, *ε’* as well as *ε’’* are smaller at area of high frequencies and increased when the frequency declined (see Figure 5a,b). At the area of low frequencies, the *ε’* and *ε’’* values are large because the SS blocking electrodes cause the accumulation of charges at the interfaces between the PEs and electrodes [54]. The *ε’* and *ε’’* reduction at the area of high frequencies is caused by the very rapid reversal period of applied EF that causes a decline in the dielectric constant [55].

The correlation between *σ_DC_* and *ε’* can be described qualitatively. The formula for ionic conductivity has been written by [56]:

(11) $$\sigma_{dc}=n\;\times q\;\times \;\mu$$

where *n* stands for the charge carrier density, *q* is 1.6 × 10^−19^ C, and *µ* stands for the mobility of the ions. From Equation (11), it is apparent that both *µ* and *n* increases when the amount of Gly is increased from 10 to 40 wt.%. It is vital mentioning that the *n* is directly interconnected to the dissociation energy *(U)* as well as *ε’* that is comprehended by the relation (*n* = *n_o_* *exp*(−*U*/*ε’K_B_T*), where *K_B_* stands for the Boltzmann constant and *T* stands for the absolute temperature. The increment in *ε’* due to the insertion of Gly causes enhancement in *σ_DC_* since *n* increased [56,57].

### 3.6. EDLC Characteristics

#### 3.6.1. Transference Number Measurement

**Figure 6:**
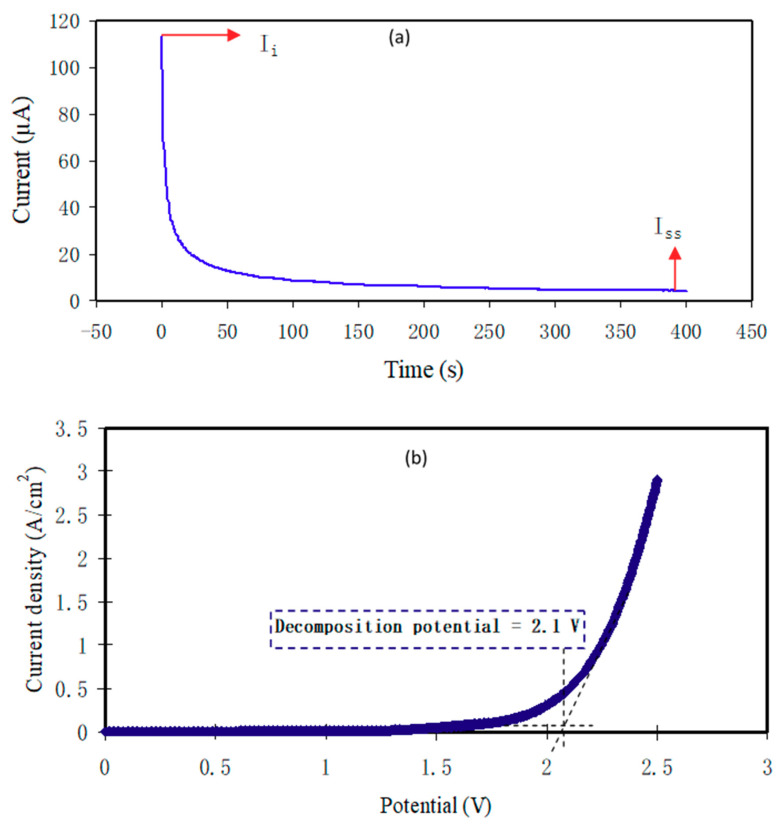
(**a**) Polarization current versus time for the PNCG-4 electrolyte and (**b**)linear sweep voltammetry (LSV) for the PNCG-4 film of PCE.

The participation of ions and electrons in the total conductivity is found by TNM measurement and analysis as shown in Figure 6a. After applying the 0.20 V, the current commences to decrease till it meets the saturation. The polarization of current in opposition to time for the utmost conducting PCE (PNCG-4) is shown in Figure 6a. The cause for the great initial current is owing to the involvement of electrons and ions at the initial stage. The cell has been polarized once it meets the steady-state; whereas the transfer of the remained current is only due to electrons. This is because the SS electrodes block the ions; however it permits the electrons to move through [58,59,60]. For this reason, SS electrodes can determine the electron transference number (*t_el_*). Ion transference number (*t_ion_*) and *t_el_* have been achieved using Equations (12) and (13):

(12) $$t_{ion}=\;\frac{I_{i}-I_{ss}}{I_{i}}$$

(13) $$t_{el}=1-t_{ion}$$

where initial and steady-state current are specified as *I_i_* and *I_ss_*, correspondingly.

Equations (12) and (13) have been performed to evaluate *t_ion_* and *t_el_*, and the receiving values of *I_i_* and *I_ss_* are 113.7 and 4.3 µA, correspondingly. The *t_ion_* and *t_el_* have been calculated to be 0.962 and 0.038, correspondingly. The *t_ion_* is of great interest since the value of *t_ion_* is very close to the ideal value. Afterward, it is recognized that ions have a key role in the mechanism of transportation in the PCE system. Shukur and Kadir [33] documented that *t_ion_* for NH_4_Cl Gly-based PE is between 0.91 and 0.98.

#### 3.6.2. Electrochemical Stability Study

The PE electrochemical stability is vital to be assessed for their use in electrochemical devices like EDLC [61]. The performance of device is displayed once there is an alert of the PCEs electrochemical stability before starting the charge–discharge cycles assessment. To preserve the PCEs, the decomposition voltage is critical. The cell setup for LSV was analogous with the examination of TNM. Figure 6b illustrates the LSV plot of PNCG-4 at 10 mV s^−1^ with a voltage range between 0 and 2.5 V. The changes in current are not clearly seen within the working electrode in the potential range between 0 and 2.15 V. Potential window is seen at 2.1 V, suggesting PCEs decomposition.

This outcome is identical with the study by Lim et al. [28] on PVA:LiClO_4_:TiO_2_ PCE with electrochemical stability window of 2.4 V. They applied the PCE in an EDLC. The electrochemical stability window of the protonic battery is typically around 1 V [2]. Therefore, the PNCG-4 decomposition voltage reveals its suitability for the use in protonic devices. Asmara et al. [62] documented that charge carrier density (*n*) impacts the decomposition voltage. This might be because of the *n* increment with the Gly insertion.

#### 3.6.3. Cyclic Voltammetry Study

**Figure 7:**
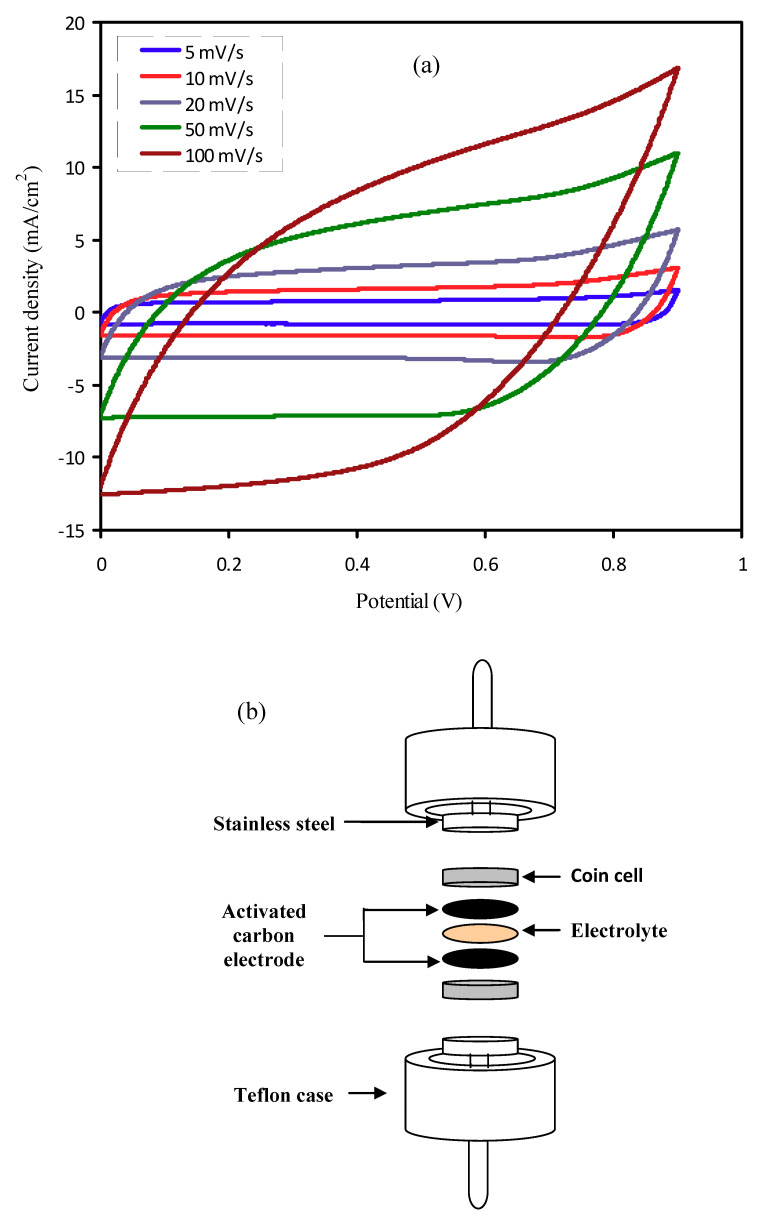
(**a**) Cyclic voltammetry (CV) curve of the developed electrical double-layer capacitor(EDLC) for the PNCG-4 film of PCE and (**b**) schematic diagram of EDLC setup for CV measurement.

Examination of cyclic voltammetry(CV) has been used to evaluate the EDLC performance using PNCG-4 electrolyte. Figure 7a shows the CV curve for EDLC at a variety of scan rates of 5, 10, 20, 50, and 100 mV s^−1^ with the schematic cell arrangement shown in Figure 7b. Peaks are not detected in the Figure 7a stating that redox reaction does not appear in the potential range between 0 and 0.9 V. This is a good indicator for the EDLCs existence [63]. It is clear from Figure 7a, that the CV shape diverges from a leaf shape to approximately rectangular in shape while the scan rate declines. As stated by Kant et al. [64], the best CV curve specifies good transportation of charges. The high scan rate departs the CV nature from a rectangular shape, which is caused by both carbon porosity and internal resistance (*ESR*), thus creating a current–voltage dependence [65].

The EDLC specific capacitance (*C_CV_*) at a variety of scan rates has been received from the CV figure by means of Equation (14):

(14) $$C_{CV}=\;\int_{V_{i}}^{V_{f}}\frac{I\left(V\right)dV}{2ma\;\left(V_{f}-V_{i}\right)}$$

The area of CV curve (*∫ I(V)dV*) has been gained using integration function in Origin 9.0 software. Here, *a* stands for the scan rate, *m* stands for the activate material mass, and *V_f_* and *V_i_* stand for the final voltage of 0.9 V and the initial voltage of 0 V, correspondingly.

The *C_CV_* values have been obtained from the CV curve using Equation (14) and are sorted in Table 5 at a variety of scan rates of 5, 10, 20, 50, and 100 mV s^−1^. The *C_CV_* value augments when the scan rate declines. Ions fill the total vacant sites within the electrodes because ions receive adequate time for the diffusion mechanism at small scan rates, causing in greater *C_CV_* values [66]. The *C_CV_* values from CV curve in the current work are higher than those in the previous study, e.g., Lewandowski et al. [67] applied PEO–KOH–H_2_O based on the solid polymer electrolyte (SPE) to an EDLC and achieved a *C_CV_* of 93 F g^−1^ at the scan rate of 2 mV s^−1^ using the CV curve for an EDLC based on active carbon. The CV curve acquired in this work is similar to that demonstrated by Zainuddin et al. [68].

### 3.7. Galvanostatic Charge–discharge Analyses

**Figure 8:**
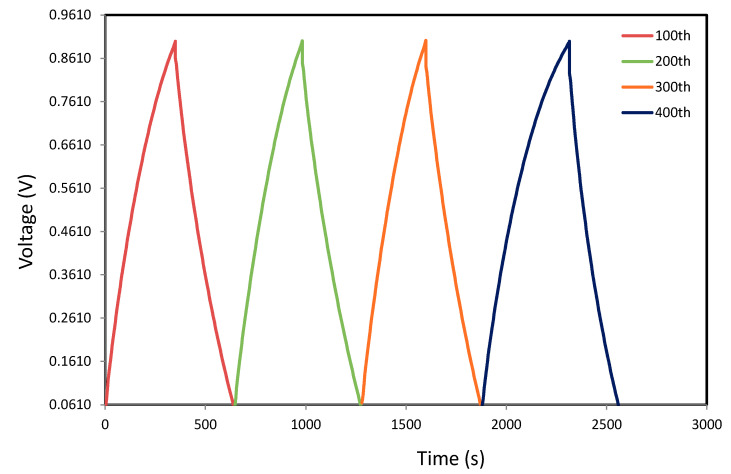
Curves of charge–discharge for the developed EDLC at 0.5 mA cm^−2^ for specified cycles.

Figure 8 explains the EDLC charge–discharge with high performance at specified cycles within 450 cycles. The discharge curves with roughly linear slope specify the EDLC capacitive behavior [66]. The discharge part with this linearity describes that the interactions among the charged pores on the surface and the ions is merely electrostatic rather than redox reaction [69]. One can note that the synthesized EDLC charge–discharge profile at 0.5 mA cm^−2^ for the specified cycles up to 450th cycles is still linear as shown in Figure 8. This is clearly signifying the presence of EDLC capacitive manner [66].

The specific capacitance (*C_d_*) using discharge curve has been computed using Equation (15):

(15) $$C_{d}=\;\frac{i}{xm}$$

**Figure 9:**
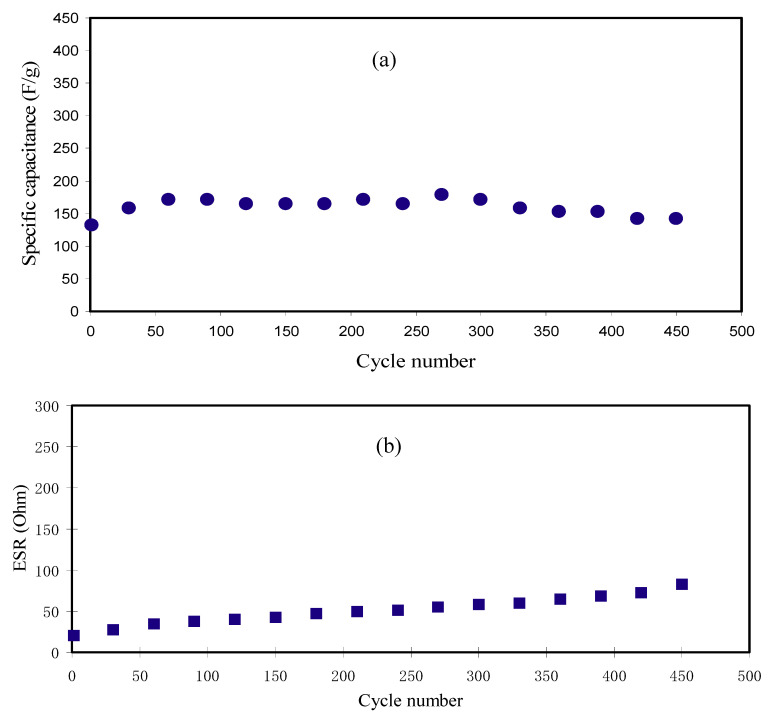
(**a**) Specific capacitance (C_d_) of the manufactured EDLC for 450 cycles and (**b**) equivalent series resistance (ESR) pattern for 450 cycles.

Here, *i* and *x* stand for the applied current and discharge part gradient, correspondingly. The *C_CV_* and *C_d_* values of the EDLC have been compared so as to examine the confidence of the outcomes. The *C_d_* has been computed by inserting the discharge curves slope value into Equation (15). Figure 9a describes the *C_d_* in opposition to number of cycles. The acquired *C_d_* for the 1st cycle is 132.749 F g^−1^. This value is very similar to the attained *C_CV_* from CV assessment (see Table 5). Therefore, the accomplished specific capacitance by the EDLC in this work is reliable. Though, Yang et al. [70] applied PVA-KOH based on the SPE to an EDLC and documented that the *C_d_* using the charge–discharge profile (112.48 F g^−1^) was bigger than the determined *C_CV_* using curve of CV (98.99 F g^−1^). The *C_d_* values in the present work subjects an increase and keeps steady with the average of 160.074 F g^−1^ beyond the 1st cycle till it completes 450th cycles. However, in previous reports, large decrease of *C_d_* was detected with raising the number of cycles [27,28]. It is illustrious that the *C_d_* of EDLCs relies on the *σ_DC_* of the PEs as described in the previous works [10,49]. The achieved *C_d_* in the present work is large in comparison with the earlier works for a variety of PEs as tabulated in Table 6.

The improvement of *C_d_* in the EDLC is ascribed to the higher *σ_DC_* of the PCE due to the addition of Gly plasticizer [36] and also the effect of the Cd(II)-complex on the enhancement of the amorphous structure. In the previous work, it was indicated that Cu(II)-complex improved the amorphous structure in the PVA-composed system [14]. According to previous report [71], the growth in the structure of amorphous is useful in local chain segmental movement because it promotes the transportation of ions and thus enhances the *σ_DC_*. Thus, ions transfer freely through the PE. Fast ion migration in the PE also encourages the adsorption of ions at the interfaces of the electrodes and PEs that provides greater *C_d_* of EDLC [27].

The situation of the EDLC electrode–electrolyte contact has been studied by means of Equation (16):

(16) $$ESR=\;\frac{V_{d}}{i}$$

where *ESR* stands for the equivalent series resistance and *V_d_* stands for the potential drop before the process of discharging.

The *V_d_* with small values (0.0205–0833.0 V) over 450 cycles in the current study designates that smaller amount of energy is lost in generating undesired heat within the mechanism of charging and discharging [23,74]. The *ESR* pattern can be seen in Figure 9b. The *V_d_* values have been gained by using Equation (16). The EDLC internal resistance is attributed as equivalent series resistance (*ESR*). Therefore, a low *ESR* is vital for EDLC purpose. As documented by Arof et al. [75], the *ESR* existence in the EDLC is because of the resistance of the current collectors, PEs, as well as the space amid the PE and the current collector. *ESR* is acquired via Equation (16) and illustrated in Figure 9b. Low *ESR* with the average of 51.05 Ω over 450 cycles designates that the PE has made an excellent contact with the electrodes that aids the movement of ions to the entrance of the pores at the electrodes [62]. Fortunately, the increase in the *ESR* of the EDLC in the current work is still insignificant within 450 cycles. The *ESR* in the present work is much lower than those reported for other EDLC devices [68,76].

EDLC energy density (*E_d_*) and power density (*P_d_*) have been calculated by means of Equations (17) and (18), respectively:

(17) $$E_{d}=\;\frac{C_{s}V^{2}}{2}$$

(18) $$P_{d}=\;\frac{V^{2}}{4m\;\left(ESR\right)}$$

where *V* stands for the applied voltage.

**Figure 10:**
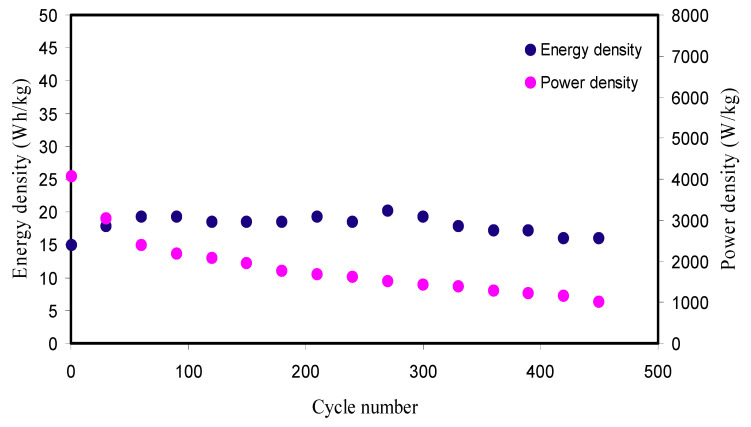
Energy density (E_d_) and power density (P_d_) of the manufactured EDLC for 450 cycles.

Figure 10 shows the *E_d_* and *P_d_* for the manufactured EDLC over 450 cycles. From Figure 10, it is perceived that the *E_d_* value using Equation (17) for the 1st cycle is 14.934 Wh kg^−1^. The *E_d_* value exhibits an augment and keeps constant with the average of 18.01 Wh kg^−1^ within the cycles beyond the 1st cycle up to 450 cycles. This implies that nearly the similar barrier of energy is subjected by ions during migration toward the surface of the AC electrodes for the processes of charge–discharge within 450 cycles. However, in previous reports, substantial decrease of *E_d_* was detected with increasing the cycles number [19,27]. They stated that the reduction in *E_d_* values within the cycles number is a result of the *ESR* increment that causes more energy loss during the mechanism of charge–discharge cycles [65,77]. The *E_d_* of the synthesized EDLC is much greater than the earlier reports with numerous PEs as recorded in Table 6. As stated by Ragone plot [78], the *E_d_* values of supercapacitors (Scs) are ranged between 0.05 and 20 Wh kg^−1^.Fortunately, the value of *E_d_* (18.01 Wh kg^−1^) within 450 cycles in the current work is very close to the battery energy density. These results indicate that the Cd(II)-complex as filler was greatly impacted on the EDLC device performance.

It is worth noting that the generated *E_d_* in the current work using PCE is higher than the provided *E_d_* of the gel supercapacitors (Scs) using gel polymer electrolyte as reported by Lee et al. [73]. They showed an ionic liquid-based polymer gel electrolyte for gel Scs but with an *E_d_* of 15.7 Wh kg^−1^ [73]. However, generally, gel Scs possesses higher *E_d_* than solid-state Scs.

The calculated *P_d_* value using Equation (18) for this work is shown in Figure 10. Upon charge–discharge for 450 cycles, the *P_d_* for the 1st cycle is 4065.040 W kg^−1^ and slightly subjected to fall up to 200th cycles with the average of 2393.437 W kg^−1^ and then kept constant with the average of 1318.917 W kg^−1^ throughout the remaining cycles beyond the 200th cycle. The *P_d_* falls at larger cycles is because of the PE depletion. Agglomerated ions after the rapidly charge–discharge mechanisms prohibit the migration of ions to the electrodes that causes the ions adsorption reduction at the interfaces of the electrodes and PEs [79]. This *P_d_* drop inclination matched with the rising *ESR* tendency in Figure 9b. The *ESR* raise at greater cycles is owing to the PE depletions, and aggregated ions after the rapid mechanism of charge–discharge cycles subsequently offer smaller *P_d_* at higher cycles [80]. The accomplished *P_d_* for the EDLC is significant as compared to the previous report (198.7 W/kg) for PVA:LiClO_4_:TiO_2_-based PCE [28]. As stated by the Ragone plot [78], Scs may possess *P_d_* up to 10^6^ W/kg. Ragone plot is essential to comprehend and distinguish among Scs, batteries, and fuel cells. This plot shows that the batteries and fuel cells are large *E_d_* systems; whereas Scs are high *P_d_* systems.

## 4. Conclusions

In conclusion, PVA:NH_4_SCN:Cd(II)-complex:Gly-based PCE were successfully fabricated and employed in EDLC application. The results of this study established that metal complex is crucial for increasing the performance of energy storage devices. The addition of Cd(II)-complex and Gly maximized the conductivity to 2.01 × 10^−3^ S cm^−1^. The XRD indicated that the electrolyte with highest DC ionic conductivity (*σ_DC_*) possesses the minimum degree of crystallinity. The FTIR spectroscopy specified that NH_4_SCN, Cd(II)-complex, and Gly have interacted with the PVA through the shifting of the FTIR bands. The FESEM route revealed that the surface morphology of the films was smooth uniform at higher Gly concentration. The trend of conductivity has been more confirmed by the study of dielectric. Transference numbers of ions (*t_ion_*) and electrons (*t_el_*) are specified to be 0.96 and 0.04, respectively, designating that the predominant carrier species are ions. LSV measurement explored that PNCG-4 electrolyte decomposed at 2.1 V, suggesting the PCE appropriateness for application in EDLC. The manufactured EDLC has been studied with CV curve and charge-discharge cycles. The rectangular shape of the CV plot designated the capacitive behavior of an ELDC. Using the charge–discharge examination, the *C_d_* and *E_d_* of the EDLC were almost constant at 160.07 F g^−1^ and 18.01Wh kg^−1^, correspondingly, for 450 cycles of charge–discharge. The low *ESR* value displayed that the EDLC exhibited superior contact among the AC electrodes and the PCEs. The EDLC had initial high P_d_ as 4065 W/Kg.

## Figures and Tables

**Table 1:** The degree of crystallinity from deconvoluted XRD analysis.

Electrolyte	Degree of Crystallinity (%)
Pure PVA	41.68
PNCG-1	13.64
PNCG-2	10.39
PNCG-3	9.38
PNCG-4	6.47

**Table 2:** The FTIR results of poly(vinyl alcohol) (PVA) and doped PVA.

Assignments	Wavenumber (cm^−1^)				
	PVA	PNCG-1	PNCG-2	PNCG-3	PNCG-4
O–H stretching	3317	3353	3361	3349	3361
C–H stretching	2913	2924	2916	2924	2928
Aromatic S–C=N stretching	–	2045	2043	2049	2047
C=O stretching	1643	1612	1611	1615	1620
C–H bending vibration of CH_2_	1415	1409	1412	1409	1418
C–H deformation	1319	1316	1313	1305	1308
–C–O– stretching	1082	1026	1037	1030	1034
C–H rocking	838	833	838	840	838

**Table 3:** The electrical equivalent circuit(EEC) fitting parameters for polymer composite electrolytes (PCEs) system at room temperature.

Sample	P_1_ (rad)	P_2_(rad)	*K*_1_ *(F^−1^)*	*K*_2_ *(F^−1^)*	*C*_1_ *(F)*	*C*_2_ *(F)*
PNCG-1	0.83	0.58	4.2 × 10^8^	2 × 10^5^	2.38 × 10^−9^	5 × 10^−6^
PNCG-2	0.87	0.47	7 × 10^6^	4.7 × 10^4^	1.43 × 10^−7^	2.13 × 10^−5^
PNCG-3	–	–	–	2.2 × 10^4^	–	4.55 × 10^−5^
PNCG-4	–	0.65	–	2.1 × 10^4^	–	4.76 × 10^−5^

**Table 4:** Achieved *σ_DC_* of the PVA:NH_4_SCN:Cd(II)-complex:Gly system at room temperature.

Designation	Composition (PVA wt.%:NH_4_SCN wt.% :Cd(II)-complex mL:Gly wt.%)	*σ_DC_* (S cm^−1^)
PNCG-1	50:50:10:10	7.21 × 10^−6^
PNCG-2	50:50:10:20	6.52 × 10^−5^
PNCG-3	50:50:10:30	1.38 × 10^−3^
PNCG-4	50:50:10:40	2.01 × 10^−3^

**Table 5:** Capacitance values from cyclic voltammetry (CV) against scan rates.

Scan Rates (mv/s)	Capacitance (F/g)
100	56.47
50	86.42
20	116.59
10	128.48
5	130.77

**Table 6:** Electrical double-layer capacitors (EDLCs) specific capacitance (*C_d_*), energy density (*E_d_*), as well as cycle numbers using dissimilar polymer electrolytes (PEs) at surrounding temperature.

Electrolyte Composition	*C_d_* (F g^−1^)	*E_d_* (Wh kg^−1^)	Cycle No.	Reference
PVA:LiClO_4_:TiO_2_	12.5	1.56	1000	[28]
PS:MC:NH_4_NO_3_:Gly	31	3.1	1000	[36]
PVA-CH_3_COONH_4_-BmImCl	28.36	2.39	500	[49]
PVA:CH_3_COONH_4_:BmImBr	21.89	1.36	500	[19]
MC:dextran:NH_4_I	79	8.81	100	[72]
PEO–KOH–H_2_O	90	–	–	[67]
PVA-KOH	112.48	10	1000	[70]
EMIM-TFSI:PVDF-HFP	51.8	15.7	3000	[73]
**PVA:NH_4_SCN: Cd(II)-complex:Gly**	**160.07**	**18.01**	**450**	**This work**

where, LiClO_4_ = lithium perchlorate, TiO_2_ = titanium dioxide, PS = potato starch, MC = methylcellulose, CH_3_COONH_4_ = ammonium acetate, BmImBr = 1-butyl-3-methylimidazolium bromide, BmImCl = 1-butyl-3-methylimidazolium chloride, NH_4_I = ammonium iodide, PEO = poly(ethylene oxide), KOH = potassium hydroxide, EMI-TFSI= 1-ethyl-3-methylimidazolium bis(trifluoromethanesulfoly) amide, PVdF= poly(vinylidene fluoride), and HFP= hexafluoropropylene.
